# Bystander Responses to a Violent Incident in an Immersive Virtual Environment

**DOI:** 10.1371/journal.pone.0052766

**Published:** 2013-01-02

**Authors:** Mel Slater, Aitor Rovira, Richard Southern, David Swapp, Jian J. Zhang, Claire Campbell, Mark Levine

**Affiliations:** 1 Institució Catalana de Recerca i Estudis Avançats (ICREA), Passeig Lluís Companys, Barcelona, Spain; 2 The Event Lab, Departament de Personalitat, Avaluació i Tractaments Psicològics Facultat de Psicologia, Campus de Mundet, Edifici Teatre Passeig de la Vall d'Hebron, Barcelona, Spain; 3 Department of Computer Science, University College London, London, United Kingdom; 4 National Centre for Computer Animation, The Media School, Bournemouth University, Poole Dorset, United Kingdom; 5 Department of Psychology, Lancaster University, Fylde College, Lancaster, United Kingdom; 6 Department of Psychology, Edge Hill University, Ormskirk, United Kingdom; 7 Department of Psychology, University of Exeter, Streatham Campus, Northcote House, Exeter, United Kingdom; George Mason University/Krasnow Institute for Advanced Study, United States of America

## Abstract

Under what conditions will a bystander intervene to try to stop a violent attack by one person on another? It is generally believed that the greater the size of the crowd of bystanders, the less the chance that any of them will intervene. A complementary model is that social identity is critical as an explanatory variable. For example, when the bystander shares common social identity with the victim the probability of intervention is enhanced, other things being equal. However, it is generally not possible to study such hypotheses experimentally for practical and ethical reasons. Here we show that an experiment that depicts a violent incident at life-size in immersive virtual reality lends support to the social identity explanation. 40 male supporters of Arsenal Football Club in England were recruited for a two-factor between-groups experiment: the victim was either an Arsenal supporter or not (in-group/out-group), and looked towards the participant for help or not during the confrontation. The response variables were the numbers of verbal and physical interventions by the participant during the violent argument. The number of physical interventions had a significantly greater mean in the in-group condition compared to the out-group. The more that participants perceived that the Victim was looking to them for help the greater the number of interventions in the in-group but not in the out-group. These results are supported by standard statistical analysis of variance, with more detailed findings obtained by a symbolic regression procedure based on genetic programming. Verbal interventions made during their experience, and analysis of post-experiment interview data suggest that in-group members were more prone to confrontational intervention compared to the out-group who were more prone to make statements to try to diffuse the situation.

## Introduction

A violent and unprovoked attack by one person on another unfolds in close view of an unrelated bystander: under what conditions will the bystander be likely to intervene to help the victim? In this paper we address the hypothesis that group affiliation between the bystander and the victim provides a powerful incentive for the bystander to try to intervene to stop the attack, or prevent harm to the victim, and in particular that this operates even though the perpetrator and victim are virtual human characters. Our experiment involved fans of an English football team, Arsenal. In one experimental condition (in-group) the fan conversed with a virtual character that was clearly an Arsenal supporter and in another condition the character was just a general football enthusiast but not an Arsenal fan (out-group). The virtual character was later threatened by a perpetrator that, in the in-group condition, specifically attacked his Arsenal affiliation. Our expectation was that based on group affiliation, those in the in-group would intervene more than those in the out-group. First we place this in the general context of studies of bystander intervention, and then describe the detailed design of the experiment and the results.

Research on the behaviour of bystanders in emergencies began with the response to the rape and murder of Kitty Genovese in New York in 1964. Social psychologists Bibb Latane and John Darley read a report on the murder in the New York Times suggesting that 38 witnesses had watched the murder unfold over 30 minutes from their apartment windows– and yet failed to intervene. In order to understand why this might have happened, they set out to create laboratory based experimental analogies of the event. They set up carefully choreographed situations in which bystanders were faced with a non-violent emergency situation while on their own or in the presence of others [1], [2]. The research led to the discovery of the ‘bystander effect’ – the idea that people are more likely to intervene on their own than in the presence of others [1]. This is one of the most reliable and robust findings in social psychology [3], [4].

However, as Cherry pointed out [5], through translating the events surrounding the Genovese murder into laboratory settings, Latane and Darley neglected some of the key features of the event. Despite the fact that the original murder involved violence by a man against a woman, subsequent experimental analogies tended to remove both the gendered nature of the attack and the violence. Although there are thousands of studies using non-violent emergency settings, it is possible to find only a few experiments that did retain violence as the emergency variable [6], [7], [8], [9], [10]. These found results that were at odds with the traditional bystander paradigm. In violent emergencies, what seemed to be most important about the likelihood of bystander intervention was not the presence of others, but rather the bystanders’ beliefs about the nature of the relationship between perpetrator and victim [9], [10]. In an experiment that did vary the number of bystanders to a violent emergency Harari et al. [6] showed that the presence of others actually enhanced the likelihood of bystander intervention in a simulated rape situation. This finding has been supported by contemporary work which presents violence to participants by means of a CCTV video link, where the presence of others is not found to inhibit helping [11] and can sometimes enhance it [12]. A recent meta-analysis by Fisher and colleagues [4] confirms that intervention behaviour in violent emergencies does not fit the traditional bystander effect explanation.

If violent emergencies are different in some way, it is important to understand the processes at work. Almost all violence research shares a similar limitation. In order to circumvent the practical and ethical problems of presenting violence in experimental settings, these experiments tend to avoid placing participants in *direct* contact with the violence itself. The only exception is the work described in [10] in which a role-play setting was used, and confederates actually staged a violent confrontation in front of naive participants who were also taking part in the role-play game. However, it is highly unlikely that contemporary ethics boards would allow this kind of design. The other studies either have the violence happening at a distance where it is possible to avoid the event [6] or present the violence as happening contemporaneously but where it can only be heard [7], [8], [9], or happening in another room where it can be seen on CCTV link [11]. This distancing of participants from the violence is required to satisfy the ethical and practical difficulties of experimental design, but may itself introduce psychological effects that interfere with the veridical nature of the situation. Imagining the violence, or having it happen in another room, is not the same as being physically where the violence erupts.

In [13] we argued that the use of immersive virtual environments (IVE) goes some way towards solving this problem, since there is mounting evidence that when people are faced with events and situations in an IVE they tend to behave and respond as if these were real [14]. IVEs portray a simulated computer generated reality at life size that is sensorially surrounding. Participants perceive this world through wide field-of-view stereo vision and sound. The form of perception involves more or less natural sensorimotor contingencies - meaning that the whole body is used for perception much as in physical reality, based at least on head-gaze direction and orientation achieved through head-tracking. This gives rise to the sensation of being in the virtual place that is depicted, a place-illusion. Additionally when there are dynamically unfolding events in the environment that personally refer to the participant, and where actions of the participant apparently cause responses in the virtual environment, this gives rise to a plausibility-illusion, meaning that events have the illusory quality of being real. When the participant has the double illusion - of being in the virtual place and where events that are happening are apparently really happening, this can give rise to behaviour and responses that are appropriate to the situation as if it were playing out in reality [15].

IVEs provide therefore a powerful tool for experimental studies in social psychology [16] and classic effects such as proxemics [17] where distances that people maintain between themselves are governed by social norms, have been reproduced several times in IVEs with respect to virtual humanoid characters [18], [19], [20]. Moreover, IVEs have been useful for experiments that would otherwise be difficult to carry out in any other way, such as the study of male risk taking in the presence of observers, specifically the differential effects of the observers being male or female [21].

Closer to the present study which focuses on responses to violence, the Stanley Milgram obedience paradigm [22] has been reproduced with IVE avoiding the ethical difficulties of deception [23], [24]. IVEs provide environments completely under control of a computer program but where people respond realistically. Every experimental condition can be exactly reproduced across trials as needed, and hence can be used for laboratory based experiments.

**Figure 1:**
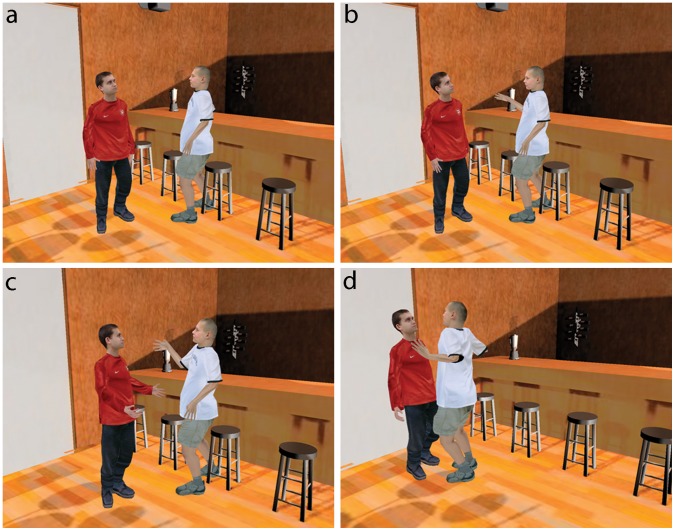
The Victim and Perpetrator. The Victim (V) is in the red shirt, with an Arsenal emblem in the in-group condition, and with a plain football shirt of the same colour in the out-group condition. The perpetrator (P) had been sitting by the bar. (a) P stood up to approach V and (b) started an argument. (c) As the argument progressed V made conciliatory statements and postures while (d) P became ever more aggressive finally pushing V violently against a wall.

It has been argued before that IVEs provide an excellent tool for the study of prosocial behaviour [25]. The experiment described in the present study is specifically concerned with the likelihood of prosocial behaviour when participants are placed in direct proximity to violent behaviour. We explore the hypothesis that the psychological relationships between bystanders and the others involved are important in bystander behaviour, in this case specifically the relationship between the bystander and the victim [12], [26], [27], [28]. The experimental conditions provide a context where it is certain that the violence between perpetrator and victim is of the same magnitude and intensity for each experimental trial. Participants (n = 40) *all supporters of the Arsenal Football Club*, entered into a virtual reality that represents a bar. A male virtual human (V) approached and conversed with them about football for a few minutes. In one condition V wore an Arsenal football shirt and spoke enthusiastically about the club (in-group condition). In a second condition V wore an unaffiliated red sports shirt, and asked questions about Arsenal without special enthusiasm, using neutral responses and displaying ambivalence about Arsenal’s prospects (out-group condition). After a few minutes of this conversation another male virtual human (P, perpetrator) who had been sitting by the bar walked over to V (victim) and started an argument that he continually escalated until it became a physical attack (Figure 1).

The main response variable was the extent to which the participant attempted to intervene during this confrontation. Interventions were verbal utterances or physical moves towards the two virtual characters and were coded from video recordings by two independent researchers (Methods). There were two binary factors *group* and *LookAt*. *Group* was whether V was in-group (Arsenal supporter) or out-group with respect to the participant. *LookAt* was whether or not occasionally during the confrontation V would look towards the participant or not (*LookAt* = ‘on’ or ‘off’). The experiment used a between-groups design, with n = 40, 10 participants allocated arbitrarily to one of the four cells of the 2×2 design. The degree of support for the Arsenal club was similar between the 4 experimental conditions (Text S1). At the end of their session they answered a questionnaire, and this was followed by an interview and debriefing. The data from two participants could not be used due to video recording failures.

## Results

### Numbers of Interventions

Table 1 shows the means and standard errors of the numbers of interventions indicating that the mean number of interventions was higher for the in-group than the out-group, but that the *LookAt* factor had no effect. Two-way analysis of variance was carried out on the response variables, the number of physical (*nPhys*) and number of verbal (*nVerbal*) interventions. ANOVA for *nPhys* indicates that the mean is greater for the in-group than for the out-group condition (P = 0.02) but with no significant differences for the *LookAt* factor and no interaction effect. However, the residual errors of the fit were strongly non-normal (Shapiro-Wilk test P = 0.0008). To overcome this problem a square root transformation was applied to *nPhys*. This resulted in the same conclusions for *group* (P = 0.016, partial η^2^ = 0.15) and no significance for *LookAt* (P = 0.297, partial η^2^ = 0.03). The normality of the residuals is improved although not ideal (Shapiro-Wilk P = 0.034). For the response variable *nVerbal* the results were similar: ANOVA of *nVerbal* on *group* and *LookAt* shows no significant interaction term, *group* has significance level P = 0.095, and for *LookAt* P = 0.228. However, again the residual errors are far from normal (SW P = 0.0008). The square root transformation gives P = 0.060, partial η^2^ = 0.10 for *group* and P = 0.112, partial η^2^ = 0.07 for *LookAt*. The residual errors are compatible with normality (SW P = 0.24).

**Table 1:** Means and Standard Errors of Numbers of Interventions.

	No. Verbal Interventions		
*Group*	*LookAt*		
	Off	On	All
Outgroup	3.9±1.4	2.0±1.3	2.9±1.0
Ingroup	6.8±1.8	4.7±1.9	5.8±1.3
All	5.4±1.2	3.4±1.2	4.4±0.8
	**No. Physical Interventions**		
Outgroup	2.8±1.1	1.8±1.0	2.3±0.7
Ingroup	6.8±2.1	6.1±2.2	6.5±1.5
All	4.9±1.3	4.1±1.3	4.5±0.9

n = 9 for each of the two Out-group cells, n = 10 for each of the two In-group cells, n = 38 in total.

The factor *LookAt* represents whether the V avatar was *programmed* to occasionally look toward the participants. Additionally, the post experience questionnaire included the statement (*VictimLooked*) “After the argument started, the victim looked at me wanting help” which was scored on a scale from 1 (least agreement) to 7 (most agreement). *VictimLooked* therefore represents the *belief* of the participants as to whether the victim *looked towards them for help*. There is no significant difference between the mean *VictimLooked* score of those who were in the group *LookAt* = ‘on’ (mean 3.3, SD = 1.8, n = 20) and those in the group *LookAt* = ‘off’ (mean 4.0, SD = 1.5, n = 20) (P = 0.12, Mann-Whitney U). Hence the response to this question was not based on the number of actual looks of the victim towards the participant, and therefore was a belief. It turns out that *VictimLooked* plays a significant role in the number of interventions.

**Figure 2:**
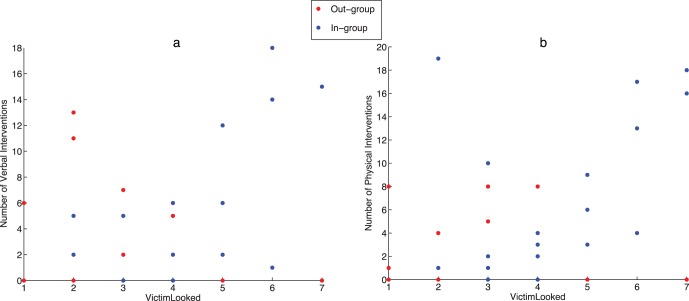
Number of interventions by *VictimLooked* and *Group*. (a) For the verbal interventions and (b) for the physical interventions.

Figure 2 shows the scatter plots of *nPhys* and *nVerbal* on the questionnaire response *VictimLooked* for the out-group and in-group. These reveal a quite different relationship in the two cases. In the case of the in-group there is a positive association between the number of interventions (verbal or physical) and the perception that the victim was looking towards the participant for help. In the case of the out-group there appears to be no relationship in the *nPhys* case and a possible negative relationship in the *nVerbal* case. Using the same strategy as above in order to obtain residual errors compatible with normality, ANCOVA of *nPhys* ^0.5^ on *group* with *VictimLooked* as a covariate shows that the slopes of the regression line are different between the in-group and out-group (P = 0.004, partial η^2^ = 0.22 for the slopes, SW P = 0.18). For the number of verbal interventions, using *nVerbal* ^0.5^ the difference in slopes between in-group and out-group is significant at P = 0.004 (partial η^2^ = 0.22 for the slope, SW P = 0.12).

These results indicate that the response to the belief that the victim was looking towards the bystander for help was different between the in-group and out-group. For those in the in-group condition the greater their belief that the victim was looking to them for help the greater the number of verbal and physical interventions. For those in the out-group condition there is no such association. These results are further corroborated using multivariate analysis of variance on the response vector (*nPhys* ^0.5^, *nVerbal* ^0.5^) (Text S2).

### Numbers of Interventions - Symbolic Regression

The previous section provided standard analyses for these types of data. Even though this revealed positive results consistent with our initial hypothesis, in this section we also employ a quite different method using symbolic regression, to throw further light on the experimental results. The purpose is to consider the relationship between the number of interventions, and the experimental factors, but now also including any possible influence of the subjective variables as elicited through the post-experience questionnaire (Table 2). Standard statistical analysis is based, amongst other things, on the assumption of linearity in the parameters. But in such a complex situation as the one under consideration, on what grounds is such an assumption valid when considering the multivariate influence of a number of factors potentially influencing bystander intervention? Symbolic regression does not rely on such linearity, being a method for discovering relationships between variables using the technique of genetic programming [29] (Text S3). It has recently been shown to be able to discover complex physical laws automatically [30], using a program called Eureqa, which was used in the analysis presented below. In the context that we apply this technique here, we consider it as a data reduction method. It allows us to succinctly represent the original data but with quite simple equations while preserving the variance in the original data. It is not a technique that can be compared with statistical significance testing, it is rather a data exploration method, that can lead to understanding of complex data, where models generated by this technique can be used for hypothesis formation in later experimental study.

**Table 2:** The Post-Questionnaire and Corresponding Variable Names.

Variable	Statement
Uncomfortable	After the argument started, I was feeling uncomfortable with the situation.
OtherSafety	After the argument started I was sometimes concerned for the safety of the man being threatened.
OwnSafety	After the argument started I was sometimes concerned for my own safety.
HelpMe	After the argument started I looked around for help.
OtherPeople	After the argument started I looked around to check in case other people might arrive to make the situation worse.
VictimLooked	After the argument started, the victim looked at me wanting help.
MoveAway	After the argument started I felt I should move away from those people.
AgressorAware	After the argument started, the aggressor was aware of me looking at him.
ShouldStopIt	After the argument started, I felt I should do something to stop it.
CouldStopIt	After the argument started, I felt I could do something to stop it.
GetOut	After the argument started I felt that I needed to get out.
Thinking	My mind started wandering and thinking about other things during the argument.

All items were presented as statements on a 1–7 Likert scale where 1 meant least agreement and 7 most agreement with the corresponding statement.

The operators that were used for the symbolic regression were: Constant, +, −, ×, /, sqrt, exp, log. The program was run for both *nPhys* and *nVerbal*. The population size (number of formulae per generation) was chosen by Eureqa as 2560. For each analysis the program was run on a 40 core cluster (see Methods) and left running for many hours until the solution set of equations stabilized. The fitness metric used was mean absolute error.

We consider first *nPhys*. The Eureqa program was left to run for more than 2000 core hours. It reported 28 equations. Each has an associated size parameter that represents the complexity of the equation (ranging from 1, least, to 53, most complex), a fitness value, the square of the correlation coefficient between the response variable and the fitted values from the equation, and the Akaike Information Criterion (AIC). The AIC is an information theoretic measure of the relative goodness of fit of a model to the data. Smaller AIC values represent better goodness of fit, taking also into account the complexity of the model. The AIC is often used in model selection procedures, as discussed extensively in [31].

The model with the smallest AICs is shown in Eq (1). Here *group* is 0 for out-group and 1 for in-group. Similarly *LookAt* is 0 for ‘off’, and 1 for ‘on’. The other variables are from the questionnaire (Table 2).
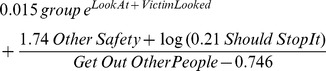
(1)


(R^2^ = 0.85, AIC = 108, Size = 26).

**Figure 3:**
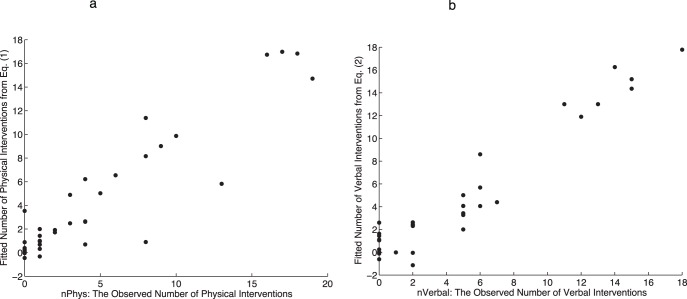
The fitted number of interventions by *VictimLooked* from Eqs. (1) and (2). (a) The fitted against observed values for *nPhys*. (b) The fitted against observed for *nVerbal*.

Figure 3a shows the relationship between the observed and fitted number of interventions based on Eq (1) (the diagram is very similar for all the top fitting equations generated). The high fitting equations all, of course, give similar results and Eq (1) is marginally preferred since it has high explanatory power (in terms of correlation) and the smallest AIC, and on the range of complexity of the models produced is about half way along the scale amongst all generated equations.

The equation shows a clear distinction between in-group and out-group. For the out-group (group = 0) the entire first term, on the left-hand side of the plus sign, vanishes (20 of the 28 equations generated have this exponential term). For the in-group (group = 1), it can be seen that *LookAt* has a very small but positive influence on the number of interventions but *VictimLooked* has a greater influence. As it ranges from 1 to 7 the number of interventions increases by 0.015*exp(*VictimLooked*), which is, for example, 2 for *VictimLooked* = 5, and 16 for *VictimLooked* = 7, other things being equal.

The second term only includes a few of the questionnaire variables. Examining this term, the number of interventions is proportional to concern about the safety of others, and the feeling that the fight should be stopped. It is inversely proportional to the feeling of wanting to get out, and the fear that other people might turn up to make things worse.

Now we turn to the number of verbal interventions *nVerbal*, and follow the same analysis. Here the genetic program ran for 1930 core hours. 28 equations were produced with size complexity ranging from 1 to 71. The equation with the lowest AIC is shown in Eq (2).
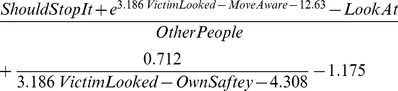
(2)


(R^2^ = 0.93, AIC = 83, Size = 29).

**Figure 4:**
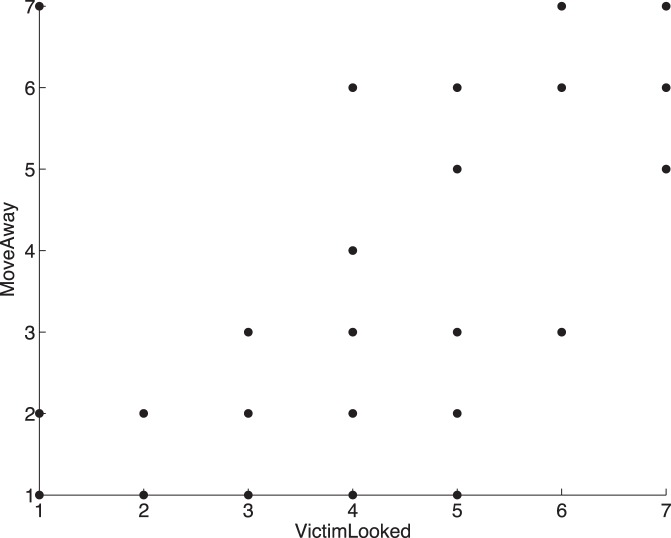
Scatter diagram of *MoveAway* against *VictimLooked*. Note the one outlying point when *VictimLooked* = 1 and *MoveAway* = 7.

As before all the high fitting equations give very similar results and we take Eq. (2) as representative. Figure 3b shows the plot of fitted by observed values over the data set for Eq. (2). Examining the equation we see this time there is no effect of *group*. The number of verbal interventions is proportional to the feeling of the need to stop the fight, and inversely proportional to the fear that other people might arrive and make things worse. Also there is a positive association with participant fears for their own safety. The most interesting variable again is *VictimLooked*, the *belief* that the V avatar was looking towards the participant for help. The variable *MoveAway* is strongly related with *VictimLooked* which must be taken into account otherwise the equations explode into huge values as *VictimLooked* increases. Figure 4 shows that there is a very strong positive correlation between these two variables (apart from 1 outlier) (r = 0.71, P = 3.3×10^−7^), with regression line *MoveAway* = −0.38+0.82*VictimLooked*. Moreover 22 out of the 28 equations include the exponential term involving these two variables. We maintain this relationship when examining the effect of *VictimLooked* on *nVerbal* rather than fixing *MoveAway* at a constant value, and taking this into account high values of *VictimLooked* are associated with a larger number of interventions.

### The Interviews

After the experimental trial there was a short interview with the participants, followed by their debriefing where the purposes of the experiment were explained. The interviews concentrated on several main questions: their feelings and responses during their experience, the extent to which they judged their responses to be realistic, factors that might have increased their intervention, and factors that drew them out of the experience. Summaries of the interviews were coded into key codes and frequency tables constructed, using the HyperResearch software [32].

We consider first the responses and feelings of participants during their experience. Table 3 shows the codes and two example sentences of each code and Table 4 the code frequencies.

**Table 3:** Codes for the Interview Questions: What feelings/responses did you have while this was happening?

Code	Example Statements
wanted to stop it	1. I felt like I would like to stop it (the confrontation) myself, basically back up the person that I was speaking to Arsenal about, protect him.
	2. I wanted to calm him down. I wanted to separate them.
uncomfortable	1. I felt very uncomfortable.
	2. I felt a little bit uncomfortable.
torn about intervening	1. I thought about intervening, do something about it, try to calm him down, but probably would have made it worse.
	2. I wanted to do something, but I felt I probably couldn’t and if I did, I might make things worse to myself. So I just tried to calm him down a little bit, but obviously he didn’t want.
would avoid confrontation	1. I would avoid confrontation.
	2. I would probably have walked out of the CAVE, like I would have done in real life if there was a problem. I was a bit afraid of talking to the man with the white shirt, in case that he would interact with me and get aggressive.
even though VR	1. I knew it was VR, and I’m quite surprise how so angry made me feel, the other guy (P), … I got to the point that I wanted touch him physically or pushing away I felt a bit frustrated I couldn’t.
	2. I was aware it was a simulation, and I was safe in that respect. I knew it was an aggressive confrontation and I think that has some impact and kind of made me a bit nervous.
anger	1. I was quite angry as well, about the way he (P) was treating him, the Arsenal fan.
	2. I’m quite surprise how so angry made me feel.
frustration	1. I got to the point that I wanted touch him physically or pushing away I felt a bit frustrated I couldn’t.
	2. …but it was a kind of frustrating I couldn’t because I tried to speak to the guy (P) and he just ignored me.
anxiety or fear	1. Very similar feelings as in real life: flustered, panic, helpless and wanting to resolve the situation and not knowing how.
	2. Frightened, I was feeling more alert, more mentally prepared for a fight.
helplessness	1. Helplessness, unable to help the Arsenal supporter.
	2. Helpless because even if was to get involved, I don’t know how useful I would be.
confrontational	1. I wanted to say I’m wearing an Arsenal shirt as well [*he was*], so your problem is with me as well, I was just criticizing his argument basically.
	2. I thought about punching the aggressor.
uninvolved	1. I felt like an observer all the time.
	2. To be honest, with VR, I was quite divorced, I was just a kind of watching.
silly or humorous	1. I thought it was a bit silly.
	2. Humorous.
concerned for or felt sorry for V	1. I was concerned for the safety for the man with the red shirt.
	2. I felt a bit sorry for the victim, a little compassion for him.
wanted to leave	1. I did feel that I wanted to leave.
	2. I wanted to leave I didn’t want to get involved.

**Table 4:** Frequencies of the Codes in Table 3.

	Frequency of statement	
Code	Out-group %	In-group %
wanted to stop it	16	18
uncomfortable	2	9
torn about intervening	11	13
would avoid confrontation	5	2
even though VR	0	7
anger	0	7
frustration	0	7
anxiety or fear	14	18
helplessness	7	7
confrontational	7	13
uninvolved	11	0
silly or humorous	5	0
concerned for or felt sorry for V	16	0
wanted to leave	7	0
**TOTAL no. of statements**	**44**	**45**

The impression from the interviews as shown in Table 4 is that those in the out-group tended to sympathize with or feel sorry for V. Also many of them wanted to just leave the situation, felt uninvolved, or a few found the situation silly. For those in the in-group it seems to be more anger and frustration that could be the driving force of their intervention, and their response was more likely to be a confrontational one. None of them felt uninvolved, found the situation funny or silly, felt sorry for V or wanted to leave. Some of the in-group expressed surprise at their own responses even though they were aware that it was virtual reality, whereas none of the out-group expressed such surprise. This fits with the fact that many of the out-group felt uninvolved and none of the in-group felt so.

Tables 5 and 6 give the results for the interview question regarding the authenticity of response in comparison with reality. We do not show the separate tables for in-group and out-group since there is no difference between them in this regard, although there is some suggestion of a difference between the *LookAt* groups. It seems that those in the *LookAt* ‘off’ group were more likely to remark on the lack of interaction, and to contrast their behaviour in virtual reality and reality. They were less likely to report their responses as being realistic. In the combined sample just over half found that their responses were realistic.

**Table 5:** Codes for the Interview Question: Were your responses realistic?

Code	example statements
realistic or quite realistic	1. I think that’s what I would do in real life.
	2. Pretty authentic. I’ve been in situation like this before, and run your mind afterwards think ‘I could have done this, I could have done that, or I should have done this’. but at that time you feel like a deer in the headlights, you are sort of frozen. You want to help, but you don’t want that guy to throw a punch on you, it’s a fine line.
lacked interaction	1. The fact that he (P) didn’t recognized me when he came over, I felt I was just watching.
	2. I behaved as in real life up to the point that I realized that there was no reaction from them.
contrasts VR and reality	1. In real life, I would try to put some distance between them and me, pub fights might be tricky, they might have weapons.
	2. I thought about it, but I wasn’t sure if they would respond to me. Anyway, in real life I would probably have not intervened. I would have been more scared in real life.
detached	1. I was completely detached.
	2. It was not authentic at all.

**Table 6:** Frequencies of the Codes in Table 5.

	Frequency of statement		
Code	LookAt off %	LookAt on %	Combined%
realistic or quite realistic	44	62	52
lacked interaction	15	5	10
contrasts VR and reality	37	24	31
detached	4	10	6
**TOTAL no. of statements**	**27**	**21**	**48**

Participants were asked what might have increased or decreased their degree of intervention. The results are shown in Tables 7 and 8. Most frequently they said that if the setup had been more interactive (i.e., the characters responding to their actions after the argument had started) then they would have been more likely to intervene. There were two other aspects that are opposed. On the one side a number of participants said that they would have been more likely to intervene if the perpetrator had become more aggressive. On the other side some participants said that they might have intervened had the perpetrator been less aggressive. Others emphasized that had the victim explicitly called for help they would have been more likely to have intervened. Another important contributory factor could have been greater rapport - for example, the victim having been a friend - or someone in need such as a child.

**Table 7:** Codes for the Interview Question: What would have made it more likely for you to intervene?

Code	example statements
	*Aspects that would have increased intervention …*
call for help	1. If the guy who was threatened would have directly spoken to me.
	2. If V would have looked at me and said something to me at some point, something like this “Can you believe this guy?”
more interactivity	1. If P would have said anything to me.
	2. If there had been a reaction from them to my first interventions.
more aggression	1. If the aggressor started punching, if the situation would become more physical.
	2. If it had turned physical, I would have stepped in. If there was another person joining, I would have definitely stepped in.
more rapport	1. If it was a child against a man or a woman against a man, or even if he is a stranger if I maybe spent a match or discuss the football before hand, so there a was a bit of relationship.
	2. If the victim was my friend, probably if there was a connection between him and I.
more realism	1. A greater degree of realism.
safety of intervention	1. Maybe if the person with the white shirt would have been less aggressive.
	2. If P would not have said that he hated Gooners, or if there were more Arsenal fans around.
	*Aspect that would have decreased intervention …*
knew it was VR	1. I knew I was in virtual reality, I wouldn’t intervene because I didn’t know if I had to.
	2. Deep down I knew it was virtual reality.

**Table 8:** Frequencies of the Codes in Table 7.

Code	Frequency of statement %
call for help	11
more interactivity	41
more aggression	16
more rapport	11
more realism	3
safety of intervention	11
knew it was VR	8
**Total no. of statements**	**37**

Finally participants were asked to talk about technical factors that drew them out of the experience. It will be seen from the video (Video S1) that, for example, there is no lip sync when the characters talk. This is very obvious when looking at the video, but barely noticeable when immersed in the environment with the life-sized characters. The combination of gesture and natural turn taking in conversation, amongst other things, are probably factors in making this glaring defect not noticeable. Only 5 out of 40 people mentioned the lack of lip sync and it was the fifth most mentioned aspect in this question. Table 9 shows the list of topics raised by the participants and the number of times they were mentioned. By far the greatest number of issues were concerned with ‘plausibility’ of the situation itself, and the technical factors tend to come down lower in the list.

**Table 9:** Frequencies of Statements in Response to the Interview Question: What factors tended to draw you out of the experience?

Topic	No. of people
No other people around	9
The pub does not look like a real English pub	7
Dialogue with the victim not realistic	7
No response from characters during the argument	6
No background noise or music	5
No mouth movement of the characters	5
Lack of sense of touch	5
Animations not smooth	5
CAVE walls and edges visible	4
Aggressor appears from nowhere	3
Mirror on top not appropriate	2
Illumination not realistic	2
Victim appears from nowhere at the start	2
Anatomical proportions of the characters	2
No bar staff	2
Clipping (part of a character going out of view)	1
Lack of sense of smell	1
The victim was too defensive	1
Victim looks ghostly due to Cave rendering	1
Lack of facial animation	1
**TOTAL No. of Statements**	**71**

## Discussion

The principal finding of this research with respect to the bystander issue is that participants in the in-group condition made more attempts at physical and verbal intervention than those in the out-group condition. Second, for those in the in-group the number of physical interventions was associated with the belief that the victim was looking towards them for help.

This second finding relies on the important distinction between the experimentally manipulated *LookAt* factor, and the questionnaire report after the experiment about how much the subjects thought that the victim was looking towards them for help (*VictimLooked*). To be clear, *LookAt* refers to whether or not *in fact* the program was making the victim sometimes look towards the participant. The second refers to the *reported belief* of the participant that the victim was looking towards him *for help*. The analysis of covariance (and Figure 2) showed that the belief that the victim was looking towards the participant for help had a differential effect depending on group. For those in the in-group condition, if they believed that the victim was looking towards them for help their number of interventions tended to be greater. For those in the out-group condition this relationship did not occur. This would not be surprising if it occurred in reality. If you consider you have group affiliation with someone and that person is looking to you for help surely this would be a more important event, more likely to move you to action, than if someone with whom you have no affiliation looks towards you for help. It is especially striking then that this also occurs also in virtual reality (where the only real people were the participants themselves): the more that the participants believed that the victim was looking towards them for help the more often did they intervene - but only those in the in-group condition.

Third, the use of symbolic regression as a data exploration method complemented and supported the results found from the classical analysis. Specifically, it provided a further demonstration that those in the in-group and out-group conditions responded quite differently to the influence of the *LookAt* factor and the *VictimLooked* response. Additionally, for those in both in-group and out-group the feeling that they should stop the argument was positively associated with an increased number of physical interventions, as was concern for the safety of the victim. However, the fear that other people might turn up to make the situation worse was inversely related to the number of physical interventions as was the feeling of wanting to get out.

The picture looks different for the number of verbal interventions. Here the group did not seem to play much role. Important factors contributing positively to the number of such interventions were the feelings by participants that they ‘should stop it’, concern for their own safety, and a strong perception that the victim was looking towards them for help. The factors that contributed negatively were the feeling of wanting to move away from the protagonists, and also the fear that other people might turn up to make the situation worse. However, in the vast majority of equations generated by the symbolic regression the belief that the victim was looking towards them for help is always together with the feeling of wanting to move away from the protagonists. These two variables have opposite effects, but in these data they are very strongly positively correlated. When *VictimLooked* is high, and *MoveAway* is held at its correlated value according to the regression relationship between them, then the number of verbal interventions becomes very high.

The out-group and in-group participants had about the same reported desire to stop the argument, the same level of feeling of being torn about intervening or not, and the same level of anxiety or fear. However, those in the in-group condition expressed greater anger and frustration, whereas those in the out-group condition were more likely to feel sorry for the victim, feel uninvolved or find the situation silly. Those in the in-group condition were more likely to react in a confrontational way compared with those in the out-group, who were looking more to defuse the situation. When we classify the verbal interventions as to whether they were more aimed at defusing the situation or more confrontational, amongst the out-group 17% were confrontational compared to 40% for the in-group, and 73% were defusing utterances compared to 60% for the in-group. These data suggest that the in-group were more likely to respond to the situation through anger and confrontation compared to the out-group, who were either less likely to become involved at all, or more likely to make verbal interventions to defuse the situation. This is not too surprising since by insulting the Arsenal affiliation of the victim in the in-group situation, the perpetrator was also of course indirectly insulting the participants who were all Arsenal supporters.

These data also suggest that physical interventions were more related to the safety of the victim, whereas verbal interventions were more related to safety of the self. The equations for the verbal interventions are more likely to include the ‘own safety’ than those for the physical interventions.

A final point regarding the ‘out-group’ is that in a sense it is not really an ‘out-group’ condition. Rather it is simply not ‘in-group’. Recalling the fact that all the participants were Arsenal supporters, for the ‘out-group’ the victim was portrayed as a football supporter of unknown affiliation (though highly unlikely to be Arsenal). The fact that there are clearly different results between the in-group and out-group condition is therefore a quite strong one: it is ‘in-group’ versus simply not ‘in-group’.

An important issue is the extent to which these findings are generalizable. We have shown an example where the group affiliation was a real one: strong supporters of a particular football team. This is unlike many laboratory based experiments where an abstract group affiliation is created for the purposes of the experiment. Our experimental manipulation involved activating the Arsenal affiliation through the virtual character V wearing an Arsenal football shirt, and talking enthusiastically about the club (in-group). The affiliation was not activated for those in the out-group condition, since V was not wearing an Arsenal shirt, and did not engage in enthusiastic conversation about the club. Our interest focused on the extent to which this activated (or not) psychological group affiliation impacted intervention behaviour. Our procedure was therefore designed to generate meaningful psychological group membership - the Arsenal fans were representative of a particular group. Our claim is that it is the perception that the victim belongs to the same group as the participant (in this context he was ‘one of us’) that leads people to be more likely to intervene. Hence our general hypothesis is that had the group identification been through some other means (social class, race, members of a tennis club, or even arbitrary groups conjured for an experiment) the results would have been similar.

It could be argued that the group of participants might have been too diverse in order to draw these types of conclusions. However, we argue that diversity of the sample is not relevant to this study. Arsenal fans are clearly made up of men, women, Londoners, working class, middle class, and people of different ethnic origins. However, the point is that under some circumstances they come to define themselves as members of the same group (in this case Arsenal fans) - and when this aspect of identity is important to them they are more likely to intervene to help a victim of violence when they think that person shares group membership with themselves in this context. Such group membership can be so powerful that it has been shown to at least temporarily cut across even racial bias in a context where group affiliation was created in a laboratory setting [33], [34]. It has further been argued with respect to the famous Milgram obedience and Zimbardo Stanford prison experiments [35] that group identification is an excellent predictor of conformity [36], [37]. For example, it was demonstrated, on the basis of the complete set of Milgram’s experiments, that the more that subjects identified with the experimenter and his causes (science, answering an important scientific problem) the more likely that they would administer the shocks. On the other hand they would be more likely to disobey the more that they identified with the Learner (representing the general community). Milgram’s original set of experiments provided a range of circumstances that led to varying degrees of identification with one of these groups (science or the community), and the degree of obedience varied accordingly.

Now we consider how our experiment could be improved. In [15] the concept of ‘plausibility’ of experiences in IVEs was introduced, referring to the illusion of participants that the virtual events are really happening (even though they know that this is not the case). It was argued that plausibility depends at least on three factors: (i) the extent to which there are events that refer personally to the participant, (ii) the extent to which the environment responds to actions of the participant, (iii) and the credibility of the scenario in terms of how much they fit expectations from a similar situation in reality. With respect to the technical setup there were no differences between in-group and out-group, and this is reflected in the fact that there are no differences in reported responses and feelings elicited through the interviews. However, the evidence does suggest (Table 6) a greater tendency for the group with *LookAt* ‘on’ to say that their responses were realistic, and for those with *LookAt* ‘off’ to mention the lack of interaction. This is consistent with (i) above.

However, an overwhelming conclusion from these data is that the plausibility of the experience would be greatly improved through more interactivity (i.e., (ii) above). Recall that there was an interactive episode at the start of the experiment, where in order to establish the in-group and out-group conditions, the eventual victim did have a conversation with the participant. However, once the argument started there was no further interaction in the sense that the virtual characters did not respond to anything that the participant said or did, except for the pre-programmed *LookAt* factor. Another aspect of plausibility that would need to be improved based on the results of this experiment is the credibility of the scenario itself (iii). As seen from Table 9 the types of factors that drew people out of the scenario were to do with the setting rather than the technical aspects of the display: no other people around in the pub, it did not look like a real English pub, and the dialogue with the victim itself not being realistic. More than 50% of the statements made in Table 9 refer to these types of general credibility, and the remainder are specific technical issues such as ‘Illumination not realistic’ or ‘Lack of facial animation’, none of which were commonly stated. By technical issues we refer to aspects of the scenario that require only programming to solve (such as the provision of lip sync). By more general credibility issues we refer to the simulation itself - aspects that require a better understanding of what needs to be there for this to be believable as a fight in a London pub.

Apart from the introduction of interactivity and other issues relating to credibility, there are several improvements for later versions of this experiment. For example, we have not said anything about the role of the social identity of the perpetrator with respect to the participant. Moreover there are clearly other issues involved - such as participant fear of being harmed by the perpetrator. This has not been considered at all, but could also be incorporated into an experiment through manipulation of the appearance of the perpetrator (for example, to look more or less menacing). Finally, future experiments will also manipulate the number of bystanders, and thus directly tackle the question of the role of the number of bystanders in intervention.

In this paper we have shown that immersive virtual reality can be usefully exploited to study the likelihood of bystander intervention in interpersonal violent incidents. The paradigm allows the investigation of what participants did do and think during an actual experience involving violence rather than their opinion of what they might do or what they think others might do - whether based on watching a video or on a verbal description of a situation [38]. Moreover we have exploited the powerful tool of genetic programming to explore these data in a deeper way than is possible with normal statistical methods, highlighted by the elegant distinction between the in-group and out-group conditions shown in Eq. (1).

Of course, there is still no proof that what participants would do in a physically real situation would match that which we find in virtual reality. However, as reported in the introduction to this paper there is evidence to suggest that people do respond realistically in IVEs. In fact since these experiments can never be carried out in reality, ultimately the question of the validity of people’s responses to the virtual situation can never be known through laboratory based experiments of any kind. However, our approach can be used in the process of constructing theories, that can then be further tested with the use of experiments in virtual reality, and moreover ultimately examine how well these theories fit what might be found in actual experiences in the field.

To conclude, we note that the findings for this type of research can also have implications for policy. For example, by creating an atmosphere where it is thought that not running away from a violent scene is the right thing to do, and by encouraging people to ask for help when they are victims of such a situation, it may be possible to engineer pro-social behaviour in specific circumstances where this is thought desirable by policy makers, and actually to manipulate the same variables to avoid it in other situations (e.g., “do not approach this man since he is considered armed and dangerous”). Here it would be a question of using the group to enforce social norms for the prevention of violent behaviour. The key to tackling the so called ‘walk–on-by’ society lies in using the power of group identification to promote social solidarity – and to persuade and empower bystanders to intervene, in situations where this is considered by the authorities to be appropriate.

## Methods

### Ethics Statement

The experiment was approved by the UCL Research Ethics Committee, and was carried out under written informed consent from each participant.

### The Virtual Reality System

A four screen projection system driven by a 5 PC cluster was used. We refer to this by generic name ‘Cave’ being the type of system described in [39]. The Cave has three 3 m×2.2 m back-projected screens: front, left, and right, and a 3 m×3 m front projection surface on the floor. The computers in the cluster contain Intel Pentium 3.2 GHz processors with 1 gigabyte of RAM and Nvidia Quadro FX 5600 graphics cards. The display resolution is 1024×768 pixels for each screen.

The participants were fitted with Crystal Eyes shutter glasses that were synchronized with the projectors, delivering active stereo at 45 Hz each eye. Head-tracking was performed with an InterSense IS-900 tracking device.

The program was written using the XVR programming platform as described in [40]. The virtual characters were animated using the Hardware Accelerated Library for Character Animation, HALCA [41].

### The Scenario

Two professional actors were hired to act the scene for the character animation motion capture. A Vicon motion capture system with 6 infrared cameras was used to capture their motions simultaneously. Sound was also recorded at the same time using Audacity software (audacity.sourceforge.net) with two wireless microphones attached to each actor. This raw data was then cleaned up, synchronized and split into pieces so that each one could be later assigned to a button on the interface to be played when needed during the study.

During the experiment the free-flowing conversation between the participant and V was achieved by operator control. A number of utterances had been recorded for V, each one making a statement or asking a question of the participant. Each such utterance was selected interactively by a hidden operator who could hear the responses of the participant. The operator sat by a computer screen, and all the phrases were represented visually as selectable buttons on the screen. When a button was selected (by point-and-click with the mouse) then V would say the phrase with a corresponding animation.

There was a defined script that the operator followed, but when the participant said something that fell outside of the script, then a number of general phrases could be selected by the operator in order to keep the conversation going in a natural way. For example, if the participant said something out of line, the operator could select a phrase such as “Totally agree with you” which would then be said by V. The overall effect for most of the time for most participants sounded as if it were a normal conversation between two people.

### Procedures and Scenario Details

40 male participants were recruited by advertisements around the UCL campus, where we specified that we needed football supporters (‘soccer’ in American usage). They were required to complete a questionnaire that asked about their favourite team in the English Premier League and how much they supported this team. We only recruited those who supported Arsenal Football Club to the level of at least 4 on a scale from 1 (not at all) to 7 (very much so). They were paid £7 ($10–12) for their participation. The experiment was approved by the UCL Research Ethics Committee.

Upon arrival at the laboratory participants were given a short questionnaire to complete that obtained information as to their English proficiency, medication, recent alcohol intake, degree of computer game playing, and past familiarity with virtual reality. Their age was obtained at the recruitment stage, in order to ensure that no one under 18 would be recruited.

After this they were given an information sheet to read, and the same information was again told to them verbally. This described the equipment that would be used. It also warned them that some people experience a degree of nausea in virtual reality systems, and that they were free to withdraw at any time without giving reasons. They were told that they would virtually visit a bar where something was to take place and that they should feel free to interact with other people there. They were warned that experience was going to involve discussion about football and the language and situation depicted was realistic. It included the statement “If you are someone who would be put off by witnessing realistic scenes that might include bad language or aggressive behaviour, then you should not take part in this experience.”

After participants had agreed to take part they were given a consent form to read and sign, and again told that they were free to leave the experiment without having to give reasons. They were then invited to take off their shoes to enter the Cave, and put on the eyeglasses.

The participants entered the virtual reality and were asked to look around and observe the scene, which was a bar of size 4.5 meters deep by 18 meters wide. The participant was then left alone in the bar having been instructed to look around for items related to football for 2 minutes. This allowed them to become familiar with the bar and accommodate to the virtual reality display including the shutter glasses and the overall brightness of the scene. After this time, a virtual character entered the scene and started a conversation with the participant by saying “You alright mate?” in the in-group version, and “Hi, how is it going?” in the out-group one.

Not every conversation was the same across all participants in each detail due to different responses by the participants. Table S1 shows two such conversations, one when V is an Arsenal football club fan (in-group) and the other when just a general football fan (out-group).

After about 2 minutes of this conversation they were interrupted by another character (P) that had been sitting by the bar, who stood up and approached V and said to him “Oy! Have you got a problem?” and then accused V of “staring” at him (Figure 1). This quickly became a strong verbal attack on V, with V remaining submissive throughout. Eventually after 140 s the P avatar started to violently push V, at which point the program ended and the participant took off the glasses and left the Cave.

The participants then were asked to complete a questionnaire about their various responses to the situation (Table 2) followed by an interview and debriefing where the purposes of the experiment were explained to them, and they were asked not to discuss it with others for 3 months in case they spoke with a future participant. They were then paid the £7 ($10) and the experimental trial was complete.

### Experimental Design

The experimental design was 2×2 between groups, with 10 participants arbitrarily assigned to each of the 4 cells. The two factors were *Group* (in-group/out-group) and *LookAt* (off/on). In-group was signified by V wearing an Arsenal football shirt, and maintaining an initial enthusiastic conversation about Arsenal. Out-group was signified V wearing a football shirt the same as for the in-group except without the Arsenal insignia, and during the conversation his responses were neutral and did not show much interest in the team (Table S1).

*LookAt* referred to whether V had been programmed to occasionally look at the participant during the confrontation or not. If ‘yes’ then 5 times during the confrontation V looked toward the participant for 3 seconds. This was possible since the head-tracking streamed continual real-time data to the computer program about the position and orientation of the participant’s head. If ‘no’ there was no particular programmed action that would lead V to look towards the participant, but this may occasionally have occurred by chance (depending on where the participant was standing at the time).

### Response Variables

Our major response variable of interest concerned the extent to which participants intervened during the confrontation. A video camera was mounted above the Cave looking down at the scenario and recorded each entire experimental trial. The video for two participants could not be analyzed, one for a participant in condition ‘out-group’ and *LookAt* ‘off’ and the other in condition ‘out-group’ and *LookAt* ‘on’. These participants were eliminated from all analysis involving counts of the number of interventions. The videos were analyzed independently by two different people, covering the time from when P first accosted V to the end. They had been instructed to count the number of verbal (*Verbal*) and physical (*Physical*) interventions.

### Video Analysis

Figure S1 shows two stills from one of the recordings - with first P at the start of the confrontation and then a moment while the participant was intervening by placing himself between V and P and raising his hand.

First one of the experimenters carried out a review of all the videos noting the number of times that the participant said something to the virtual characters (variable *nVerbalApprox*) and the number of physical interventions - meaning the number of times that the participant moved closer to the characters or reached out towards them (*nPhysApprox*). Second and independently someone not associated with the research, and not knowing its purposes was hired to carry out a complete video analysis using the ELAN system and as paid work (www.lat-mpi.eu/tools/elan).

The instructions were to record the total number of utterances during the relevant period (*nVerbalElan*) and also the number of physical interventions (*nPhySElan*). The instructions for the physical interventions were to regard as an intervention or an attempt at intervention an action accompanied with verbal intervention or reaching out to either of the avatars. When not accompanied by these it was considered intervention if the participant was walking with purpose towards the avatars or was followed by another form of physical or verbal intervention. Walking or stepping towards the avatars was not considered to be intervention if the participant took a step forwards, backwards, or to the left or right when far from the avatars, if they walked or stepped forwards and this was followed by them standing passively watching the avatars or when they were walking around the environment and the avatars and appeared to be simply investigating the surroundings.

Although the procedures used for the approximate and ELAN based intervention recordings were not the same the results are strongly correlated. Table S2 shows the correlation coefficients between the various measures of intervention. The approximate and ELAN based methods were consistent, with highly significant positive correlations.

Since the ELAN based method was carried out by someone not involved in the research team and more thorough, with the notion of ‘intervention’ more rigorously and conservatively defined, we base all analysis on this, so that *nPhys* = *nPhysElan*, and *nVerbal* = *nVerbalElan*.

## Supporting Information

Figure S1 **A still from a video recording from above.** (a) The participant can be seen near the centre with the victim to his left, and the perpetrator to his right. (b) The participant has stepped between the victim and perpetrator standing in front of the latter and raising his hand.
(TIF)

Table S1 **Examples of Conversations between the Virtual Character V, and participant S.**
(DOCX)

Table S2 **Pearson Correlation Coefficients Between the Intervention Variables.**
(DOCX)

Text S1 **Degree of Support for the Arsenal Football Club.**
(DOCX)

Text S2 **MANOVA for the Number of Physical and Verbal Interactions.**
(DOCX)

Text S3 **Symbolic Regression.**
(DOCX)

Video S1 **The experimental scenario for the in-group condition.**
(MP4)

## References

[1] Darley JM, Latané B (1968) Bystander Intervention in Emergencies - Diffusion of Responsibility. Journal of Personality and Social Psychology 8: 377–383. 5645600 10.1037/h0025589

[2] Latané B, Rodin J (1969) A lady in distress: Inhibiting effects of friends and strangers on bystander intervention. Journal of Experimental Social Psychology 5: 189–202.

[3] Latané B, Nida S (1981) 10 Years of Research on Group-Size and Helping. Psychological Bulletin 89: 308–324.

[4] Fischer P, Krueger JI, Greitemeyer T, Vogrincic C, Kastenmüller A, et al (2011) The bystander-effect: A meta-analytic review on bystander Intervention in dangerous and non-dangerous emergencies. Psychological Bulletin 137: 517–537. 21534650 10.1037/a0023304

[5] Cherry F (1995) The “stubborn particulars” of social psychology: essays on the research process: Routledge.

[6] Harari H, Harari O, White RV (1985) The reaction to rape by American male bystanders. The Journal of social psychology 125: 633–658. 10.1080/00224545.1985.9712039 3831611

[7] Schwartz SH, Gottlieb A (1976) Bystander reactions to a violent theft: Crime in Jerusalem. Journal of Personality and Social Psychology 34: 1188–1199. 1003323 10.1037//0022-3514.34.6.1188

[8] Schwartz SH, Gottlieb A (1980) Bystander anonymity and reactions to emergencies. Journal of Personality and Social Psychology 39: 418–430. 7431204 10.1037//0022-3514.39.3.418

[9] Shotland RL, Straw MK (1976) Bystander response to an assault: When a man attacks a woman. Journal of Personality and Social Psychology 34: 990–999.

[10] Borofsky GL, Stollak GE, Messe LA (1971) Sex differences in bystander reactions to physical assault. Journal of Experimental Social Psychology 7: 313–318.

[11] Fischer P, Greitemeyer T, Pollozek F, Frey D (2006) The unresponsive bystander: Are bystanders more responsive in dangerous emergencies? European Journal of Social Psychology 36: 267–278.

[12] Levine M, Crowther S (2008) The responsive bystander: How social group membership and group size can encourage as well as inhibit bystander intervention. Journal of Personality and Social Psychology 95: 1429–1439. 19025293 10.1037/a0012634

[13] Rovira A, Swapp D, Spanlang B, Slater M (2009) The use of virtual reality in the study of people's responses to violent incidents. Frontiers in Behavioral Neuroscience 3: 59 doi:10.3389/neuro.08.059.2009. 20076762 10.3389/neuro.08.059.2009 PMC2802544

[14] Sanchez-Vives MV, Slater M (2005) From Presence to Consciousness through Virtual Reality. Nature Reviews Neuroscience 6: 332–339. 15803164 10.1038/nrn1651

[15] Slater M (2009) Place Illusion and Plausibility can lead to realistic behaviour in immersive virtual environments. Philos Trans R Soc Lond 364: 3549–3557. 19884149 10.1098/rstb.2009.0138 PMC2781884

[16] Blascovich J, Loomis J, Beall A, Swinth K, Hoyt C, et al (2002) Immersive virtual environment technology as a methodological tool for social psychology. Psychological Inquiry 13: 103–124.

[17] Hall ET (1973) The hidden dimension. Leonardo: 94–94.

[18] Wilcox LM, Allison RS, Elfassy S, Grelik C (2006) Personal space in virtual reality. ACM Transactions on Applied Perception (TAP) 3: 412–428.

[19] Llobera J, Spanlang B, Ruffini G, Slater M (2010) Proxemics with Multiple Dynamic Characters in an Immersive Virtual Environment ACM Transactions on Applied Perception (TAP) 8: Article 3.

[20] Bailenson J, Blascovich J, Beall A, Loomis J (2003) Interpersonal Distance in Immersive Virtual Environments. Personality and Social Psychology Bulletin 29: 819–833. 15018671 10.1177/0146167203029007002

[21] Frankenhuis WE, Dotsch R, Karremans JC, Wigboldus DHJ (2010) Male physical risk taking in a virtual environment. Journal of Evolutionary Psychology 8: 75–86.

[22] Milgram S (1974) Obedience to Authority: McGraw-Hill.

[23] Slater M, Antley A, Davison A, Swapp D, Guger C, et al (2006) A virtual reprise of the Stanley milgram obedience experiments. PLoS ONE 1: e39 doi:10.1371/journal.pone.0000039. 17183667 10.1371/journal.pone.0000039 PMC1762398

[24] Cheetham M, Pedroni AF, Antley A, Slater M, Jáncke L (2009) Virtual milgram: empathic concern or personal distress? Evidence from functional MRI and dispositional measures. Frontiers in Human Neuroscience 3. 10.3389/neuro.09.029.2009 PMC2769551 19876407

[25] Gillath O, McCall C, Shaver PR, Blascovich J (2008) What can virtual reality teach us about prosocial tendencies in real and virtual environments? Media Psychology 11: 259–282.

[26] Levine M, Cassidy C, Brazier G, Reicher S (2002) Self-categorization and bystander non-intervention: Two experimental studies. Journal of Applied Social Psychology 32: 1452–1463.

[27] Levine M, Thompson K (2004) Identity, place, and bystander intervention: Social categories and helping after natural disasters. Journal of Social Psychology 144: 229–245. 15168427 10.3200/SOCP.144.3.229-245

[28] Levine M, Prosser A, Evans D, Reicher S (2005) Identity and emergency intervention: How social group membership and inclusiveness of group boundaries shape helping behavior. Personality and Social Psychology Bulletin 31: 443–453. 15743980 10.1177/0146167204271651

[29] Koza JR (1992) Genetic programming: on the programming of computers by means of natural selection: The MIT press.

[30] Schmidt M, Lipson H (2009) Distilling free-form natural laws from experimental data. Science 324: 81–85. 19342586 10.1126/science.1165893

[31] Burnham KP, Anderson DR (2002) Model Selection and Multimodel Inference: A Practical Information-Theoretic Approach: Springer.

[32] Hesse-Biber S, Dupuis P, Kinder TS (1991) HyperRESEARCH: A computer program for the analysis of qualitative data with an emphasis on hypothesis testing and multimedia analysis. Qualitative Sociology 14: 289–306.

[33] Kurzban R, Tooby J, Cosmides L (2001) Can race be erased? Coalitional computation and social categorization. PNAS 98: 15387–15392. 11742078 10.1073/pnas.251541498 PMC65039

[34] Van Bavel JJ, Cunningham WA (2009) Self-categorization with a novel mixed-race group moderates automatic social and racial biases. Personality and Social Psychology Bulletin 35: 321–335. 19098257 10.1177/0146167208327743

[35] Zimbardo P (2007) The Lucifer effect: Understanding how good people turn evil. Random House Inc, New York.

[36] Reicher SD, Haslam SA, Smith JR (2012) Working Toward the Experimenter Reconceptualizing Obedience Within the Milgram Paradigm as Identification-Based Followership. Perspectives on Psychological Science 7: 315–324. 26168469 10.1177/1745691612448482

[37] Haslam SA, Reicher SD (2012) Contesting the ‘‘Nature’’ Of Conformity: What Milgram and Zimbardo’s Studies Really Show. PLoS Biol 10: e1001426. 23185132 10.1371/journal.pbio.1001426 PMC3502509

[38] Banyard V (2008) Measurement and Correlates of Prosocial Bystander Behavior: The Case of Interpersonal Violence. Violence and Victims 23: 83–97. 18396583 10.1891/0886-6708.23.1.83

[39] Cruz-Neira C, Sandin DJ, DeFanti TA, Kenyon RV, Hart JC (1992) The CAVE: audio visual experience automatic virtual environment. Communications of the ACM 35: 64–72.

[40] Tecchia F, Carrozzino M, Bacinelli S, Rossi F, Vercelli D, et al (2010) A flexible framework for wide-spectrum vr development. Presence: Teleoperators and Virtual Environments 19: 302–312.

[41] Gillies M, Spanlang B (2010) Comparing and evaluating real-time character engines for virtual environments. PRESENCE: Teleoperators and Virtual Environments 19: 95–117.

