# Supplementary figures and images for: Bystander Responses to a Violent Incident in an Immersive Virtual Environment
Source: PLoS One. 2013 Jan 2;8(1):e52766. doi: 10.1371/journal.pone.0052766 (PMC3534695; doi:10.1371/journal.pone.0052766)

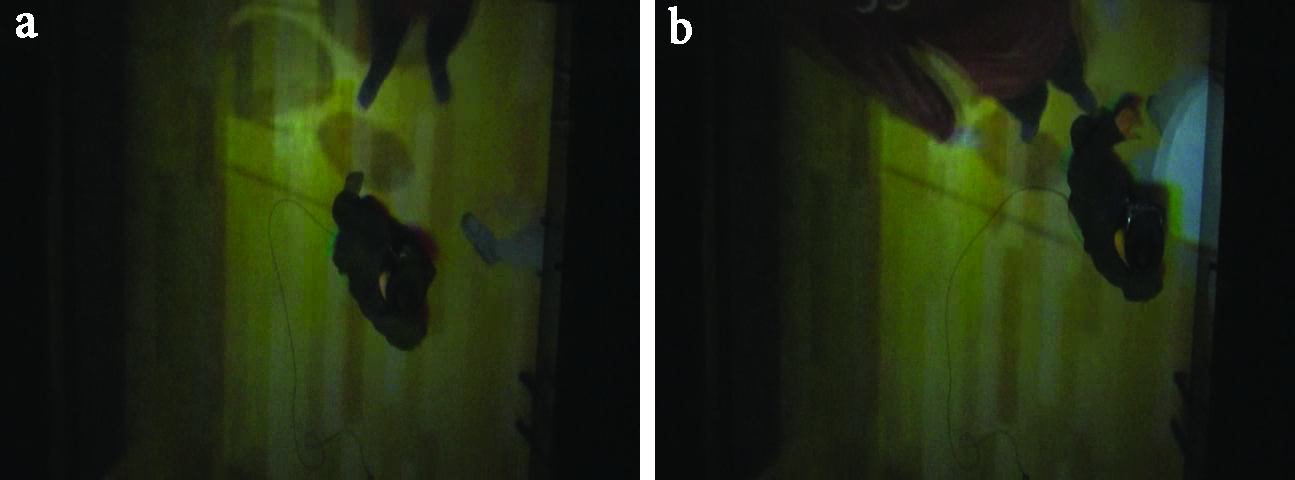

Supplement: Figure S1 — A still from a video recording from above. (a) The participant can be seen near the centre with the victim to his left, and the perpetrator to his right. (b) The participant has stepped between the victim and perpetrator standing in front of the latter and raising his hand. (TIF) [file pone.0052766.s001.tif]
