# Supplementary material for: Bystander Responses to a Violent Incident in an Immersive Virtual Environment
Source: PLoS One. 2013 Jan 2;8(1):e52766. doi: 10.1371/journal.pone.0052766 (PMC3534695; doi:10.1371/journal.pone.0052766)
Supplement: Table S1 — Examples of Conversations between the Virtual Character V, and participant S. (DOCX) [file pone.0052766.s002.docx]

**Supporting Table S1**

Examples of Conversations between the Virtual Character V, and participant S.

| **Ingroup** | **Outgroup** |
| --- | --- |
| V: You alright mate? | V: Hi, how is it going? |
| S: Hello | S: It’s alright, how are you? |
| V: Where are you from? | V: Where are you from? |
| S: Poland | S: London, I’m North London |
| V: Good. You Arsenal, yeah? | V: You Arsenal yeah? |
| S: Yeah. | S: yeah, yeah, you got it |
| V: Get yu! What do you think of the team last year? | V: I see. How did they do last season in the Premier League? |
| S: It was alright I suppose. It wasn’t too bad. | S: not too well, but this is…this will be our season. |
| V: When did you go to see a match? | V: You kidding me right? |
| S: It was last week, on Tuesday, I went to the Emirates. | S: Well I think they’ve got a good chance. Yeah, we’re doing alright, I think it’ll be our season. What team do you support? |
| V: Not too bad. Who is your favourite player? | V: Do you think they’ve got any chance of winning anything in the next couple of years? |
| S: Van Persie, I suppose | S: Well, as I said, yeah.. definitely, we were in three competitions and… |
| V: I love Fabregas, see? I’ve got it on my shirt, so.. | V: Are you sure? |
| S: nice! | S: Well, I can’t be sure, but I’m pretty confident |
| V: What do you think our chances are this season? | V: Well, current team doesn’t seem to be as good as it used to be: Henry, Vieira, Pires… Do you think they were right to get rid of Henry? |
| S: Not sure with van Persie injured it will be difficult I suppose, but we could still do it | S: I think he was getting quite old. In a way… |
| V: I’ll pray for that! We definitely gotta get something, like five years ago: Henry, Vieira, Pires.. that was a hell of a team! Do you think we were right to get rid of Henry? | V: If you say so. |
| S: yeah, well, he’s past his best, isn’t he… | S: …in a way he probably wanted to leave, he’s done well with Barcelona. But, I mean, we’ve got Fabregas, Arshavin. it’s a good team… |
| V: Anyway, sure, we got other good players, but don’t you think we can find a better goalkeeper? | V: Don’t you think they can find a better goalkeeper? |
| S: I’m not sure, I think Almunia is alright actually… | S: Yeah, of course, I mean, you know… at the same time Alumina is not too bad and there are better keepers, but I’m happy with him |
|  | V: How many times did they win the Champions League so far? |
|  | S: Yeah, I mean, but, yeah, I mean, you know… we’ve got a lot of chances… |
| (At this point the conversation was interrupted by the perpetrator avatar) | |
