# Supplementary material for: Bystander Responses to a Violent Incident in an Immersive Virtual Environment
Source: PLoS One. 2013 Jan 2;8(1):e52766. doi: 10.1371/journal.pone.0052766 (PMC3534695; doi:10.1371/journal.pone.0052766)
Supplement: Table S2 — Pearson Correlation Coefficients Between the Intervention Variables. (DOCX) [file pone.0052766.s003.docx]

**Supporting Table S2**

Pearson Correlation Coefficients Between the Intervention Variables

|  | physicalApprox | verbalElan | physicalElan |
| --- | --- | --- | --- |
| verbalApprox | 0.67 | 0.84 | 0.82 |
| physicalApprox |  | 0.46 | 0.71 |
| verbalElan |  |  | 0.74 |

All P < 0.0000, except between physicalApprox and verbalElan where P = 0.0037.
