# Supplementary material for: Bystander Responses to a Violent Incident in an Immersive Virtual Environment
Source: PLoS One. 2013 Jan 2;8(1):e52766. doi: 10.1371/journal.pone.0052766 (PMC3534695; doi:10.1371/journal.pone.0052766)
Supplement: Text S1 — Degree of Support for the Arsenal Football Club. (DOCX) [file pone.0052766.s004.docx]

# Supporting Text S2

## MANOVA for the Number of Physical and Verbal Interactions

In the main text we have carried out individual analyses of variance for the response variables *nPhys* and *nVerbal*. These response variables are highly correlated (r = 0.74, P < 0.00005), and therefore a multivariate analysis of variance (MANOVA) with the joint response vector (*nPhys*, *nVerbal*) should also be taken into account.

The Doornik-Hansen test (1) rejects the hypothesis of bivariate normality (P < 0.00005) as would be expected from the two univariate cases. Following the same strategy as in the univariate case using a square root transformation on each of the variables gives a result compatible with bivariate normality (P = 0.45).

MANOVA of (*nPhys*^0.5^, *nVerbal*^0.5^) on *group* and *LookAt* gives a significance level for *group* of P = 0.057, and for *LookAt* P = 0.282 using Wilks’ lambda. Including the covariate *VictimLooked* results in a significant interaction term between *VictimLooked* and *group* (Wilks’ lambda, P = 0.023), showing that just was found in the univariate cases the relationship between the response vector and *VictimLooked* is different between the in-group and out-group conditions. For those in the in-group the greater the belief that the victim was looking to them for help the greater the tendency for physical and verbal interactions, which was not the case for those in the out-group.

# Reference

1. Doornik JA & Hansen H (2008) An omnibus test for univariate and multivariate normality. *Oxford Bulletin ofEconomics and Statistics* 70:927–939.
