# Supplementary material for: Bystander Responses to a Violent Incident in an Immersive Virtual Environment
Source: PLoS One. 2013 Jan 2;8(1):e52766. doi: 10.1371/journal.pone.0052766 (PMC3534695; doi:10.1371/journal.pone.0052766)
Supplement: Text S3 — Symbolic Regression. (DOCX) [file pone.0052766.s006.docx]

# Supporting Text S3

## Symbolic Regression

Here we outline the method of symbolic regression. For full details see (1) below. Given a response variable (y) a number of explanatory variables (x_1_,…,x_k_), and a set of mathematical operators or functions (e.g., +, −, ×, ⁄, log, exp), a population of valid formulae over the x_i_ using these operators and functions is randomly generated. Each formula is evaluated over the data, and the result compared with the observed value of y. From this a fitness function is evaluated which indicates how well the formula explains the variation in y. From these fitness values a new generation of formulae is constructed by combining formulae selected at random with probability of selection proportional to fitness (‘mating’), and also by allowing for rare random ‘mutations’ (small changes in a formula). Formulae are selected for ‘mating’ probabilistically such that higher fitness values confer a higher chance of selection. As new generations evolve in this way, each successive generation has higher average fitness, and the process can stop once a set of formulae has been found that well explains the variation of y as functions of the x_i_. (Of course there may be no satisfactory functions, if indeed there is no relationship between the x_i_ and y).

1. Koza JR (1992) *Genetic programming: on the programming of computers by means of natural selection* (The MIT press).
